# Active Contours Driven by Multi-Feature Gaussian Distribution Fitting Energy with Application to Vessel Segmentation

**DOI:** 10.1371/journal.pone.0143105

**Published:** 2015-11-16

**Authors:** Lei Wang, Huimao Zhang, Kan He, Yan Chang, Xiaodong Yang

**Affiliations:** 1 Medical Imaging Department, Suzhou Institute of Biomedical Engineering and Technology, Chinese Academy of Sciences, Suzhou, Jiangsu, China; 2 Radiology Department, The First Hospital of JiLin University, Changchun, JiLin, China; UC Santa Barbara, UNITED STATES

## Abstract

Active contour models are of great importance for image segmentation and can extract smooth and closed boundary contours of the desired objects with promising results. However, they cannot work well in the presence of intensity inhomogeneity. Hence, a novel region-based active contour model is proposed by taking image intensities and ‘vesselness values’ from local phase-based vesselness enhancement into account simultaneously to define a novel multi-feature Gaussian distribution fitting energy in this paper. This energy is then incorporated into a level set formulation with a regularization term for accurate segmentations. Experimental results based on publicly available STructured Analysis of the Retina (STARE) demonstrate our model is more accurate than some existing typical methods and can successfully segment most small vessels with varying width.

## Introduction

Active contour models [1–4] have become very popular in the past few decades, and widely used in a wide range of problems including image segmentation and computer vision, which dynamically deforms object contours based on a predefined energy functional from image information and can, by minimizing this functional, yield smooth and closed boundary contours of the desired objects with sub-pixel accuracy [5–7]. These models can be coarsely categorized into two kinds: edge- [8–11] and region-based models [12–14].

Edge-based active contour models [15, 16] typically utilize image gradients as a driving force to identify object boundaries and attract the contour toward object boundaries. These models can be capable of extracting the boundaries of the desired objects in high-contrast images with clear and strong contour information. However, they may suffer from boundary leakage problem in the presence of image noises, weak contrast and intensity inhomogeneity, which in general occurs in a variety of medical images with varying contrast. In addition, they also cannot correctly find the boundaries of the desired objects if initial curve placement is far away from object boundaries due to the local nature of image gradients. These limitations of edge-based models have restricted their application range and in turn promoted the development of region-based active contour models, which are first introduced by Chan and Vase [13] (called the CV model), and have been subjected to more and more attentions.

Region-based models [17–20] tend to model the foreground and background regions of a given image to guide the motion of contour curve based on the assumption that image intensities are statistically homogeneous in each region, which means that the whole image can be divided into multiple image regions with different statistical properties of image intensities. Therefore, these models are robust against initial curve placement and insensitivity to image noises without using image gradients. However, they may produce erroneous segmentation results, especially in some cases where the desired objects cannot be easily distinguished using statistical information of image intensities, or have an expensive computational cost [16, 21]. These drawbacks, together with the complex problem of keeping a tradeoff among all of weighting parameters, have made these models barely useful for certain small objects, like blood vessels in retinal images.

Recently, a variety of region-based active contour models has been proposed ceaselessly by using local intensity statistical information [22–24] for more accurate image segmentation, especially in the presence of intensity inhomogeneity. Li et al. [25] improved the CV model using regularized distance and a local binary fitting (LBF) model to alleviate these problems caused by intensity inhomogeneity. This method is, to some extent, able to deal with intensity inhomogeneity using local intensity information, but sensitive to initialization and fails to extract the boundaries of object with low contrast. Wang et al. [26] proposed an active contour model driven by local Gaussian distribution fitting (LGDF) energy, which described the local image intensities by Gaussian distributions with different means and variances. The means and variances of local intensities are considered as spatially varying functions to identify the differences between the foreground and background regions. Hence, it has exhibited certain capability of handling intensity inhomogeneity and image noises, and of distinguishing regions with different intensity variances. However, it, using solely local intensity information, may fail to extract completely the desired objects.

In this paper, a novel region-based model is proposed by taking multiple image features rather than single intensity information into account simultaneously to construct a Gaussian distribution fitting energy for accurate image segmentation, called Multi-feature Gaussian distribution fitting (MGDF) model. Specifically, image intensities and their corresponding 'vesselness values' from local phase-based vesselness enhancement [27] are used to construct this fitting energy in the neighborhood of each pixel, and this energy is incorporated into a level set formulation with a regularization term for image segmentations, and evaluated based on publicly available retinal images [28] for the extraction of blood vessels.

The remainder of this paper is organized as follows: Section Background reviews several existing typical active contour models, and then a novel model is introduced in section Methods where local phase-based enhancement and a novel energy functional are presented in detail. Finally, this novel model is evaluated completely in Section Experimental results and analysis, followed by the discussions and conclusions.

## Background

There is a variety of region-based active contour models proposed in last few years, but only a few typical models are widely used and improved in image segmentations with promising accuracy. In this section, we will review several typical region-based active contour models for the benefit of the reader.

### CV Model

The CV model is first proposed by Chan et al. [13] base on the assumption that images to be segmented are simplified into multiple regions where image intensities are statistically homogeneous. Let Ω ⊂ ℜ^2^ be the 2D image domain, and *I*: Ω ⊂ ℜ be a given gray image, for each pixel point *x* in image domain Ω, the CV model can be expressed by:

$$\begin{array}{l} E^{CV}=\lambda_{1}{\int_{inside(C)}\left|I\left(x\right)-c_{1}\right|}^{2}H\left(\phi \left(x\right)\right)dx\\ +\lambda_{2}{\int_{outside(C)}\left|I\left(x\right)-c_{2}\right|}^{2}\left(1-H\left(\phi \left(x\right)\right)\right)dx+\upsilon \int \left|\nabla H\left(\phi \left(x\right)\right)\right|dx \end{array}$$ (1)

where *C* is the needed contour curve, who can be represented by the zeros level set of Lipschitz function *ϕ*(*x*): Ω ⊂ ℜ. *c*_1_ and *c*_2_ are the average intensities of the image *I*(*x*) inside and outside *C*. ∇*H*(·) is the gradient of Heaviside function *H*(·), which is usually approximated by $H\left(\phi \right)=\frac{1}{2}\left[1+\frac{2}{\pi }\text{arctan}\left(\frac{\phi }{\varepsilon }\right)\right]$ with a small positive constant *ε* to roughly specify the internal and external of *ϕ*. For simplification purposes, we define *H*_1_(*ϕ*) = *H*(*ϕ*) and *H*_2_(*ϕ*) = 1-*H*(*ϕ*) for the internal and external of *ϕ*. *λ*_1_, *λ*_2_ and *υ* are weighting parameters for intensity-based and length penalty terms, respectively. According to the assumption mentioned above, *c*_1_ and *c*_2_ should be quite different due to the statistical homogeneity of image intensities from different regions specified by *C*. When image intensities are severe inhomogeneous, *c*_1_ may be approximately equal to *c*_2_, causing the model to lose of the capability of identifying the foreground and background regions. In addition, this model solely utilizes the global information of image to drive the motion of curve contour and ignores the local information [29] around a neighborhood of each pixel point.

### LBF Model

To overcome the disadvantages of the CV model, the LBF model [25] is proposed by replacing the global information with the local information of image, which can be given by:

$$\begin{array}{l} E^{LBF}=\lambda_{1}\int \left[{\int_{inside(C)}K_{\sigma }\left(x-y\right)\left|I\left(y\right)-f_{1}\left(x\right)\right|}^{2}H_{1}\left(\phi \left(x\right)\right)dy\right]dx\\ +\lambda_{2}\int \left[{\int_{outside(C)}K_{\sigma }\left(x-y\right)\left|I\left(y\right)-f_{2}\left(x\right)\right|}^{2}H_{2}\left(\phi \left(x\right)\right)dy\right]dx+R\left(\phi \left(x\right)\right) \end{array}$$ (2)

$$R\left(\phi \left(x\right)\right)=\upsilon \int \left|\nabla H\left(\phi \left(x\right)\right)\right|dx+\mu \int \frac{1}{2}{\left(\left|\nabla \phi \left(x\right)\right|-1\right)}^{2}dx$$ (3)

where *K*_*σ*_(·) is a Gaussian kernel with standard deviation *σ*; *R*(·) is a regularization term to penalize the length of contour curve and the deviation from a signed distance function [10, 17], whose weighting parameters are *υ* and *μ*, respectively. *f*_1_(*x*) and *f*_2_(*x*) are spatially varying functions to locally approximate the intensities inside and outside the contour curve, which are quite different from the two constants *c*_1_ and *c*_2_ in the CV model due to the localization properties introduced by *K*_*σ*_(·). It plays a key role in highlighting the differences between the foreground and background regions with intensity inhomogeneity. Therefore, the model can alleviate these problems caused by intensity inhomogeneity and achieve satisfactory results. However, this model typically relies on the initial curve placement so as to avoid the local minimums of the energy functional. Furthermore, it is not sufficient enough to use solely *f*_1_(*x*) and *f*_2_(*x*) in energy functional for accurate image segmentation, especially in the presence of image noises and intensity inhomogeneity.

### LGDF Model

To accurately segment the desired objects, the LGDF model [26] is proposed by using more complex statistical characteristics of local intensities, which characterizes local intensity information via partition of neighborhood defined in a circular window, resulting in the local fitting energy being expressed as:

$$\begin{array}{l} E^{LGDF}=-\iint \omega \left(x-y\right)\text{log}p_{1,x}\left(I\left(y\right)\right)H_{1}\left(\phi \left(x\right)\right)dydx\\ -\iint \omega \left(x-y\right)\text{log}p_{2,x}\left(I\left(y\right)\right)H_{2}\left(\phi \left(x\right)\right)dydx+R\left(\phi \left(x\right)\right) \end{array}$$ (4)

where the pixel point *x* is used to define the local circle region, which is divided into *N* disjoint sub-regions ${\left\{\Omega_{i}\right\}}_{i=1}^{N}$. *p*_*i*,*x*_(I(*y*)) denotes a posteriori probability of intensity *I*(*y*) specified by a pixel point *y* in the *ith* sub-region, whose spatial weighting is given by *ω*(*x*-*y*) relying on the distance between *x* and *y*, and they can be given respectively by:

$$p_{i,x}\left(I\left(y\right)\right)=\frac{1}{\sqrt{2\pi }\sigma_{i}\left(x\right)}\text{exp}\left(-\frac{{\left(u_{i}\left(x\right)-I\left(y\right)\right)}^{2}}{2\sigma_{i}{\left(x\right)}^{2}}\right)$$ (5)

$$\omega \left(x-y\right)=\frac{1}{a}\text{exp}\left(-\frac{{\left|x-y\right|}^{2}}{2\tau^{2}}\right)$$ (6)

where *u*_*i*_(*x*) and *σ*_*i*_(*x*) are local intensity means and standard deviation respectively. *a* is a constant such that ∫ω(·) = 1 in the local neighborhood of the point *x*, *τ*>0 is a scale parameter.

**Fig 1:**
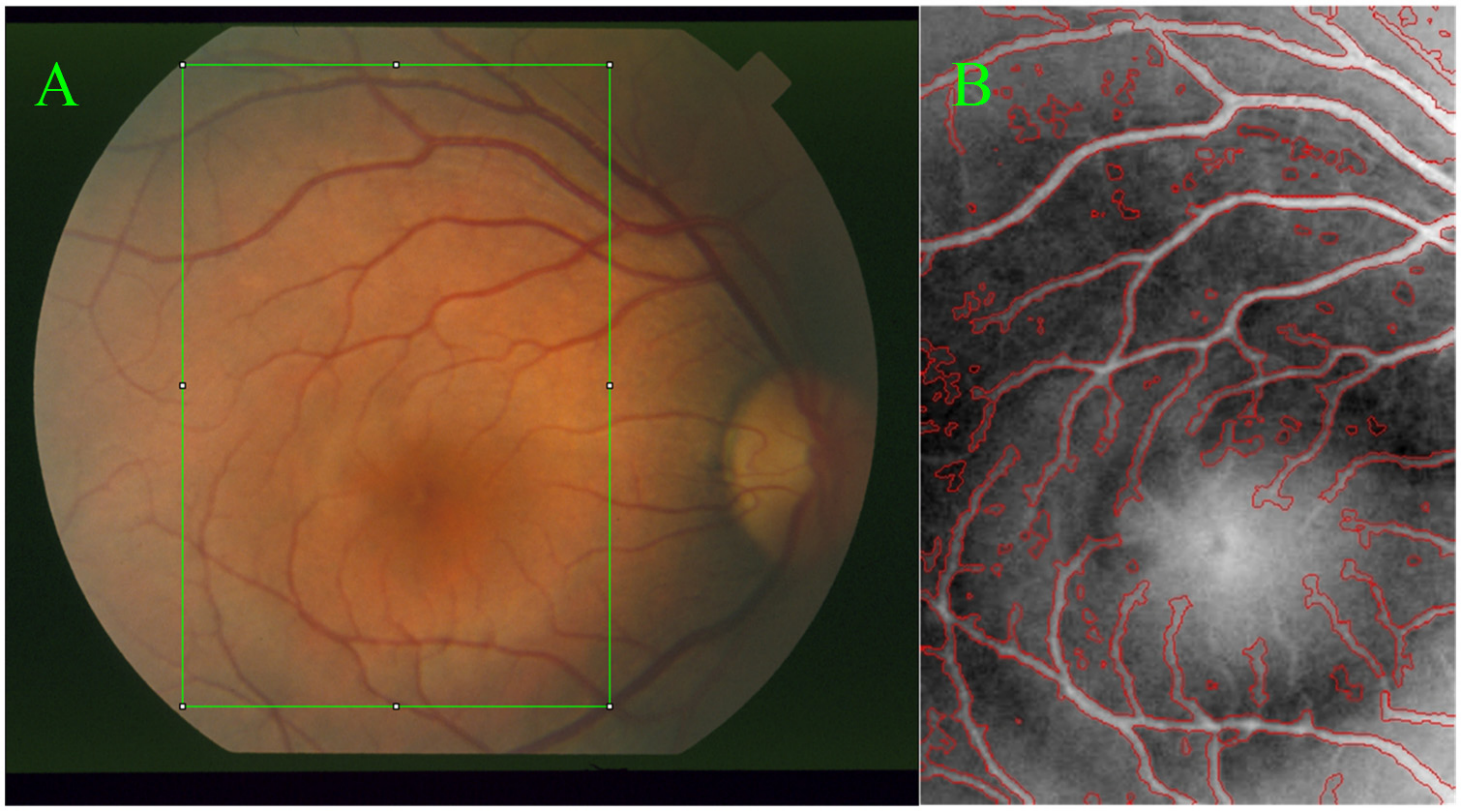
Retinal image and blood vessel extraction by active contour [26]. Image (A) is a randomly chosen region in a retinal image and (B) is the segmentation results of the LGDF model without ideal segmentations pointed by black circles.

Using local intensity means and variances, this model can achieve a relatively accurate segmentation results in the presence of image noises and intensity inhomogeneity. Besides, it is insensitive to the initial curve placement. However, it may not be adequate in certain cases where more image information rather than single intensity information needs to be considered effectively for image segmentations, especially for small vessels in retinal images. For example, this model is used to extract the vessels in the region specified by a green rectangle shown as in Fig 1(A), and its corresponding segmentation result with red color is given in Fig 1(B), where several small vessels cannot be segmented adequately, but the background pixels have been already segmented incorrectly, confirmed by black circle regions. This means that the LGDF model is not adequate for the extraction of these small vessels where the intensity inhomogeneity is quite severe. To segment accurately these blood vessels, the other image information besides image intensities has to be taken into account simultaneously.

## Methods

To identify correctly and extract completely small vessels, active contour models have to alleviate these problems caused by intensity inhomogeneity by using more image information, which is generally obtained by different feature descriptors [27, 30] and characterizes the natures of certain objects. In this section, local phase-based vesselness enhancement filter [27] is chosen to extract vesselness features and viewed as a probability-like estimate of vesselness features, called 'vesselness value' in this paper. Larger vesselness value indicates the underlying object is more likely to be a vessel structure. Therefore, these vesselness values together with image intensities can be used to define a novel fitting energy for the extraction of vessels, which will be introduced respectively in the following.

### Local phase-based vesselness enhancement

Local phase [27] is, as an important local feature, derived from quadrature filters under the concept of monogenic signals, which can be viewed as a complex filter pair in the spatial domain, and the real and imaginary parts correspond to line and edge filters, respectively. Hence, the angle *θ* between the real and imaginary parts can act as an important indicator of local features for line and edge information of an image, and has been extensively used for edge detection, symmetry analysis, and vesselness enhancements with promising results.

In the specific implementation of local phase, quadrature filters usually utilize a pair of even and odd filters with phase difference of π/2, denoted respectively by $E_{m}^{j}$ and $O_{m}^{j}$ at scale *m* and orientation *j*. For each pixel point *x* of an image *I*, the filter response $q_{m}^{j}$ is given by $q_{m}^{j}=e_{m}^{j}\left(x\right)+o_{m}^{j}\left(x\right)\cdot i$ with $e_{m}^{j}\left(x\right)=I\left(x\right)*E_{m}^{j}$ and $o_{m}^{j}\left(x\right)=I\left(x\right)*O_{m}^{j}$, where * denotes a convolution operation and $i=\sqrt{-1}$ is an imaginary unit. To obtain an orientation invariant phase map, it is necessary to replace the imaginary part $o_{m}^{j}$ with its absolute value so that $\bar{q_{m}^{j}}=e_{m}^{j}+\left|o_{m}^{j}\right|\cdot i$, and combine all responses of different directions to yield a single response at each scale, defined as $q_{m}=\sum_{j=1}^{J}\bar{q_{m}^{j}}$ with *J* directions. By combining the responses from all of scales, the overall response *P* is given below:

$$P=\frac{\sum_{m=1}^{M}q_{m}{\left|q_{m}\right|}^{\beta }}{\sum_{m=1}^{M}{\left|q_{m}\right|}^{\beta }}$$ (7)

where *M* is the number of scales and *β*≥1 is a weighting parameter. To make the phase map more regular, the response *P* needs to be normalized with a small positive number *α* to produce a final vesselness map, which can be given by:

$$LP=\frac{P\cdot \left|P\right|}{P^{2}+\alpha^{2}}$$ (8)

**Fig 2:**
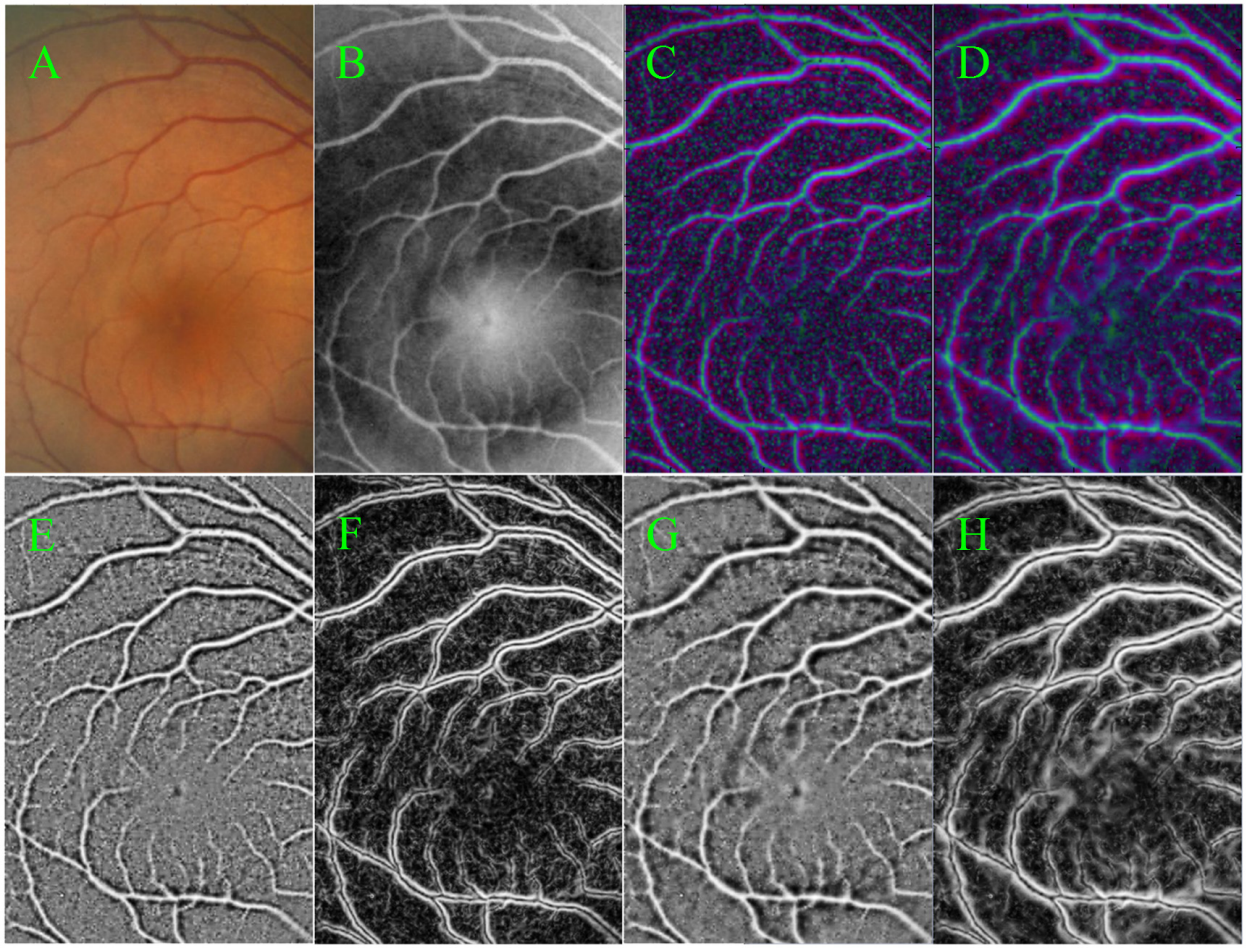
The results of the local phase-based vesselness enhancement. (A) is a randomly chosen retinal image region; (B) is the green channel of (A); (C) and (D) are the normalized results at scales 2 and 3, respectively, whose real and imaginary parts are given by (E) ~ (H)

The effects of local phase-based enhancement on retinal images are shown in Fig 2, where a variety of small vessels is highlighted clearly regardless of intensity inhomogeneity. Among of Fig 2, (A) is a given retinal image region for vesselness enhancements, the green channel of (A) is shown in (B). The normalized enhancement results of (B) are shown in Fig 2(C) and 2(D) with scales of 1 and 2, respectively. From these enhanced images, we can find that the positions of blood vessels are highlighted from the background and the enhancement effects are not greatly different at different scales, which also can be seen in their corresponding real and imaginary parts shown in Fig 2(E)–2(H), respectively. Moreover, images (E) and (G) stress the positions of vessels, which are used as a vesselness map; while images (F) and (H) stress the edges of vessels. This vesselness map has a positive value inside the vessels but a negative value in the background, and has a zero value at the edge of the line structures, which can provide an important guide for the extraction of blood vessels.

### Multi-feature Gaussian Distribute Fitting (MGDF)

Inspired by the LGDF model, a novel local fitting energy is defined by using image intensities and their corresponding 'vesselness values' from the vesselness map, which are viewed as two independent random variables to extend the LGDF model in the hope of improving its performances of image segmentation. This energy can be calculated around the neighborhood of point *x* by:

$$\begin{array}{l} E_{x}^{MLGDF}=-\int \omega \left(x-y\right)\text{log}\left(p_{1,x}{\left(I\left(y\right)\right)}^{\lambda_{1}^{I}}p_{1,x}{\left(V\left(y\right)\right)}^{\lambda_{1}^{V}}\right)dy\\ -\int \omega \left(x-y\right)\text{log}\left(p_{2,x}{\left(I\left(y\right)\right)}^{\lambda_{2}^{I}}p_{2,x}{\left(V\left(y\right)\right)}^{\lambda_{2}^{V}}\right)dy \end{array}$$ (9)

$$p_{i,x}\left(I\left(y\right)\right)=\frac{1}{\sqrt{2\pi }\sigma_{i}^{I}\left(x\right)}\text{exp}\left(-\frac{{\left(u_{i}^{I}\left(x\right)-I\left(y\right)\right)}^{2}}{2\sigma_{i}^{I}{\left(x\right)}^{2}}\right)$$ (10)

$$p_{i,x}\left(V\left(y\right)\right)=\frac{1}{\sqrt{2\pi }\sigma_{i}^{V}\left(x\right)}\text{exp}\left(-\frac{{\left(u_{i}^{V}\left(x\right)-V\left(y\right)\right)}^{2}}{2\sigma_{i}^{V}{\left(x\right)}^{2}}\right)$$ (11)

where *p*_*i*,*x*_(*I*(*y*)) and *p*_*i*,*x*_(*V*(*y*)) denote the posteriori probability of *I*(*y*) and *V*(*y*) in the intensity image and the vesselness map, located at the pixel point *Y* in the *ith* sub-region. $\lambda_{i}^{I}$ and $\lambda_{i}^{V}$ are weighting parameters for intensity- and vesselness-based terms, respectively. $u_{i}^{I}\left(x\right),\sigma_{i}^{I}\left(x\right)$ and $u_{i}^{V}\left(x\right),\sigma_{i}^{V}\left(x\right)$ are local means and standard deviations in the neighborhood of the point *x* for intensity image and vesselness map, respectively. For the whole image region, this energy can be given by:

$$E^{MLGDF}=\int_{\Omega }\left(\sum_{i=1}^{N}\int_{\Omega_{i}}-\omega \left(x-y\right)\text{log}\left(p_{i,x}{\left(I\left(y\right)\right)}^{\lambda_{i}^{I}}p_{i,x}{\left(V\left(y\right)\right)}^{\lambda_{i}^{V}}\right)\right)H_{i}\left(\phi \left(x\right)\right)dydx$$ (12)

To obtain smoother contour curves and more accurate image segmentations, this energy functional needs to be regularized by penalizing its length and deviation from a signed distance function. Furthermore, to keep fine details of the boundaries of the desired objects, we introduce the ϒ-neighborhood term to penalize the presence of isolated connected components in the segmented images according to the previous studies [31], which can be given by:

$$\Gamma =\int \left(\frac{\gamma^{\kappa }}{\gamma^{\kappa }+\phi {\left(x\right)}^{\kappa }}\right)dx$$ (13)

where *γ* and *κ*≥1 are scale parameters for a local neighborhood and the sensitivity of the local neighborhood, respectively. To combining all of these penalty terms, the complete regularization term can be rewritten as:

$$R^{\prime }\left(\phi \left(x\right)\right)=\upsilon \int \left|\nabla H\left(\phi \left(x\right)\right)\right|dx+\mu \int \frac{1}{2}{\left(\left|\nabla \phi \left(x\right)\right|-1\right)}^{2}dx+\eta \int \left(\frac{\gamma^{\kappa }}{\gamma^{\kappa }+\phi {\left(x\right)}^{\kappa }}\right)dx$$ (14)

where *η* is the weighting parameter for the ϒ-neighborhood term. Adding the new regularization term and setting *N* = 2, the entire energy of the MGDF model can be given by:

$$E^{MLGDF}=\int_{\Omega }e_{1}\left(x,y\right)H_{1}\left(\phi \left(y\right)\right)dx+e_{2}\left(x,y\right)H_{2}\left(\phi \left(y\right)\right)dx+R^{\prime }\left(\phi \left(y\right)\right)$$ (15)

$$e_{i}\left(x,y\right)=\int_{\Omega i}-\omega \left(x-y\right)\text{log}\left(p_{i,x}{\left(I\left(y\right)\right)}^{\lambda_{i}^{I}}p_{i,x}{\left(V\left(y\right)\right)}^{\lambda_{i}^{V}}\right)dy$$ (16)

By calculus of variations, it can be shown that the parameters $u_{i}^{I}\left(x\right)$, $u_{i}^{V}\left(x\right)$, $\sigma_{i}^{I}\left(x\right)$ and $\sigma_{i}^{V}\left(x\right)$ that minimize *E* ^*MLGDF*^ satisfy the following Euler—Lagrange equations [32–34]:

$$\int \omega \left(x-y\right)\left(u_{i}^{I}\left(x\right)-I\left(y\right)\right)H_{i}\left(\phi \left(y\right)\right)=0$$ (17)

$$\int \omega \left(x-y\right)\left(u_{i}^{V}\left(x\right)-V\left(y\right)\right)H_{i}\left(\phi \left(y\right)\right)=0$$ (18)

$$\int \omega \left(x-y\right)\left(\sigma_{i}^{I}{\left(x\right)}^{2}-{\left(u_{i}^{I}\left(x\right)-I\left(y\right)\right)}^{2}\right)H_{i}\left(\phi \left(y\right)\right)=0$$ (19)

$$\int \omega \left(x-y\right)\left(\sigma_{i}^{V}{\left(x\right)}^{2}-{\left(u_{i}^{V}\left(x\right)-V\left(y\right)\right)}^{2}\right)H_{i}\left(\phi \left(y\right)\right)=0$$ (20)

From Eq (17) to Eq (20), we can obtain

$$u_{i}^{I}\left(x\right)=\frac{\int \omega \left(x-y\right)I\left(y\right)H_{i}\left(\phi \left(y\right)\right)dy}{\int \omega \left(x-y\right)H_{i}\left(\phi \left(y\right)\right)dy}$$ (21)

$$u_{i}^{V}\left(x\right)=\frac{\int \omega \left(x-y\right)V\left(y\right)H_{i}\left(\phi \left(y\right)\right)dy}{\int \omega \left(x-y\right)H_{i}\left(\phi \left(y\right)\right)dy}$$ (22)

$$\sigma_{i}^{I}{\left(x\right)}^{2}=\frac{\int \omega \left(x-y\right){\left(u_{i}^{I}\left(x\right)-I\left(y\right)\right)}^{2}H_{i}\left(\phi \left(y\right)\right)dy}{\int \omega \left(x-y\right)H_{i}\left(\phi \left(y\right)\right)dy}$$ (23)

$$\sigma_{i}^{V}{\left(x\right)}^{2}=\frac{\int \omega \left(x-y\right){\left(u_{i}^{V}\left(x\right)-V\left(y\right)\right)}^{2}H_{i}\left(\phi \left(y\right)\right)dy}{\int \omega \left(x-y\right)H_{i}\left(\phi \left(y\right)\right)dy}$$ (24)

Minimization of the energy *E* ^*MLGDF*^ in Equation. Eq (15) with respect to *ϕ* can be achieved by solving the gradient descent flow equation:

$$\begin{array}{l} \frac{\partial \phi }{\partial t}=-\delta \left(\phi \right)\left(\lambda_{1}^{I}e_{1}^{I}-\lambda_{2}^{I}e_{2}^{I}+\lambda_{1}^{V}e_{1}^{V}-\lambda_{2}^{V}e_{2}^{V}\right)+\upsilon \delta \left(\phi \right)div\left(\frac{\nabla \phi }{\left|\nabla \phi \right|}\right)\\ +\mu \left(\nabla^{2}\phi -div\left(\frac{\nabla \phi }{\left|\nabla \phi \right|}\right)\right)-\eta \frac{\kappa \gamma^{\kappa }}{{\left(\gamma^{\kappa }+\phi {\left(x\right)}^{\kappa }\right)}^{2}}\phi^{\left(\kappa -1\right)} \end{array}$$ (25)

$$e_{i}^{I}\left(x\right)=\int \omega \left(x-y\right)\left[\text{log}\left(\sqrt{2\pi }\right)+\text{log}\left(\sigma_{i}^{I}\left(y\right)\right)+\frac{{\left(u_{i}^{I}\left(x\right)-I\left(y\right)\right)}^{2}}{2\sigma_{i}^{I}{\left(y\right)}^{2}}\right]dy$$ (26)

$$e_{i}^{V}\left(x\right)=\int \omega \left(x-y\right)\left[\text{log}\left(\sqrt{2\pi }\right)+\text{log}\left(\sigma_{i}^{V}\left(y\right)\right)+\frac{{\left(u_{i}^{V}\left(x\right)-V\left(y\right)\right)}^{2}}{2\sigma_{i}^{V}{\left(y\right)}^{2}}\right]dy$$ (27)

where $\delta \left(\phi \right)=\frac{1}{\pi }\frac{\varepsilon }{\varepsilon^{2}+\phi^{2}}$ is the derivative of H(*ϕ*).

## Experimental Results and Analysis

### Experimental Data

To objectively evaluate the segmentation performances of our model, the public and available image datasets (STructured Analysis of the Retina, STARE) [28] are used for segmentation experiments (S1 File), which can be available at http://www.ces.clemson.edu/~ahoover/stare/. This dataset contain 20 color photographic images of the fundus, 10 of which show evidence of pathology, acquired by a Topcon TRV-50 fundus camera (Topcon, Tokyo, Japan). They have the same size of 605×700, along with two different manual segmentations generated by clinical experts working in the field of retinal image, and the first manual segmentations are used as ground truth for quantitative analysis.

### Implementation details

In the course of vesselness enhancement, local phase-based filter was implemented by setting the center frequency to 5π/7, the bandwidth to 2 octaves, the size of filter to 15×15, and scales to 3 respectively, which were recommended by the previous studies [27] according to the nature of retinal images. After obtaining the desired enhancement images, our model is implemented based on image intensities and their corresponding 'vesselness values' from vesselness map with a level set framework, where the partial and temporal derivatives in Eq (25) are discretized as central and forward differences, respectively. And then the level set function *ϕ* is initialized as a binary step function which takes a negative constant value -*c*_0_ inside a region Ω_1_ and a positive constant value *c*_0_ outside, which we set to 2 in this paper. Spatial weighting *ω*(·) can be truncated as a (2*τ*+1)×(2*τ*+1) mask for the computational efficiency of our model, where *τ* is the smallest odd number no less than 2*σ*. Unless otherwise specified, other parameters in our experiments are set as follows:*σ* = 3, $\lambda_{1}^{I}=\lambda_{1}^{V}=1.05$,$\lambda_{2}^{I}=\lambda_{2}^{V}=1.0$, time step Δ*t* = 0.1, *υ* = 0.00065×255×255 and *μ* = *η* = 1 by default.

### Evaluation Criteria

For purposes of quantitative evaluations, segmentation results are compared with their corresponding standard segmentation results and the results by other methods [25, 26] in terms of sensitivity (Se), specificity (Sp), accuracy (Acc) and the area under a receiver operating characteristic curve (Auc) [35], which can be given respectively by:

$$Se=\frac{tp}{tp+fn}$$ (28)

$$Sp=\frac{tn}{tn+fn}$$ (29)

$$Acc=\frac{tp+tn}{tp+fp+tn+fn}$$ (30)

$$Auc=\frac{Se+Sp}{2}$$ (31)

where *tp*,*fn*,*tn* and *fp* denote the true positive (correctly identified vessel pixels), false positive (incorrectly identified vessel pixels), true negative (correctly identified background pixels) and false negative (incorrectly identified background pixels), respectively. Among of the four measures, Se and Sp demonstrate the effectiveness of segmentation algorithms: the former for the desired pixels with positive values while the latter for the undesired pixels with negative values. Acc indicates the overall segmentation performance, and Auc reflect the tradeoffs between the Se and Sp according to [35]. In addition, vessel segmentation can be in essence referred as to an imbalanced data classification problem, where vessel pixels are much less than the background pixels. In such a case, the final performances of a method are mainly reflected by Acc and Auc. Paired t-test on these evaluation measures is implemented using the SPSS version 21.0 (SPSS Inc., Chicago, IL, USA). A p value of 0.05 is considered statistically significant, according to these papers [36], in order to evaluate the segmentation performances of different methods.

### Experimental Results

**Fig 3:**
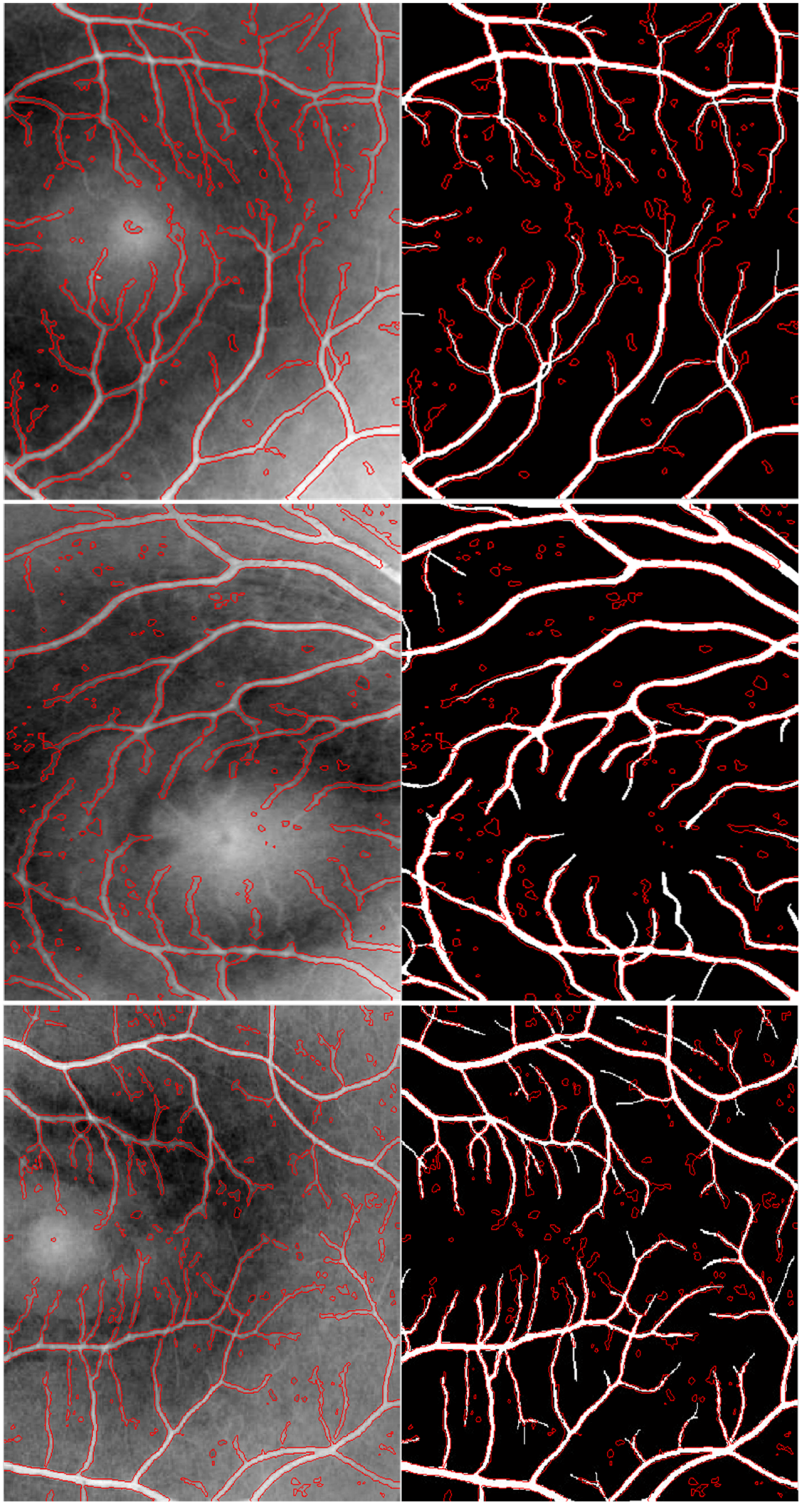
The segmentation results by the proposed model in contrast to the manual segmentations. The first row corresponds to the segmentation results by the proposed model based on three randomly chosen retinal image regions; and the second row shows the differences between these segmentation results and their corresponding manual segmentations.

According to the implementation details above, the segmentation results of the MGDF model for three randomly chosen retinal image regions are shown in Fig 3, where the first column corresponds to the segmentation results highlighted by red contour curves, and the second column to the differences between these segmentation results and their corresponding manual segmentations. From Fig 3, it is easy to find that our proposed model is able to accurately extract most of the desired contours of blood vessels, including relatively small parts, some of which cannot be identified visually in original images due to the presence of severe intensity inhomogeneity. Moreover, these obtained contour curves can successfully overlap their corresponding manual segmentations, as shown in the second column in Fig 3. This indicates clearly that our proposed model can be at least comparable to manual segmentation in performance when ignoring the subtle parts of blood vessels. On the other hand, Fig 3 also shows that the segmentation results of our model contain lots of isolated components caused probably by image noises, and they are slightly different from the manual segmentations which present more details for small vessels. However, these components account for a relatively small portion of the whole segmentation results. Therefore, we can roughly reach a conclusion that our proposed model is competent for the extraction of vessels with varying width.

**Fig 4:**
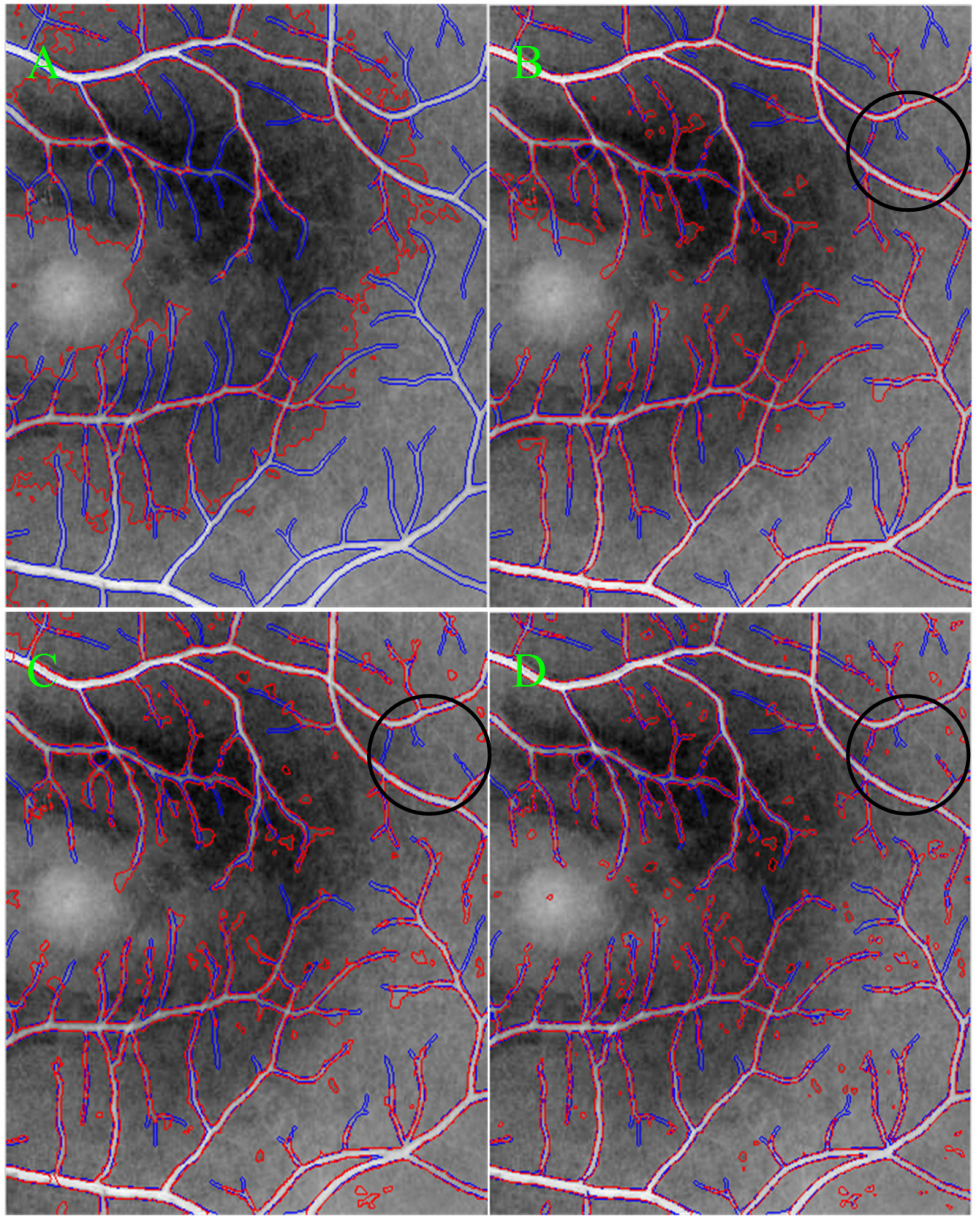
The segmentation results of different models with red color are compared with the manual segmentation with blue color. (A) ~ (D) correspond to the CV, LBF, LGDF and MGDF models, respectively.

Fig 4 also illustrates the performance differences when comparing our proposed model with several existing typical active contour models (*i*.*e*., the CV, LBF and LGDF models), where blue and red colors correspond to the contours of blood vessels obtained by manual and different models (*i*.*e*., the CV, LBF, LGDF and our models), respectively. These obtained contour curves by different models can be compared among one another, relative to manual segmentation curve. And it is easy to find that our proposed model can extract more vessels, with varying width, than the other models, which can be seen in the region specified by black circles. As for three other active contour models, their segmentation results are greatly different as shown in Fig 4(A)–4(C), respectively. Specifically, the segmentation result of the CV model, as shown in Fig 4(A), is rough and incorrect because it just involves intensity information in image global region, without considering local image information in the neighborhood of each pixel. When the local information of each pixel is considered during image segmentations, the segmentation results can be greatly improved as shown in Fig 4(B) and 4(C) obtained by the LBF and LGDF models. Both of them are able to extract easily blood vessels with strong contrast with high accuracy. However, they cannot accurately segment a variety of small vessels due to the presence of intensity inhomogeneity and image noises. In addition, the LBF model tends to extract some background information in the neighborhood of vessels with small width, while the LGDF model tends to segment several isolated components.

To compare our model with the most recent methods, the quantitative segmentation results are given in Table 1 in terms of Se, Sp, Acc and Auc. This table shows that the MGDF model has the best performances among these four active contour models with the biggest values of Acc and Auc; while the CV model has the worst performances with the lowest values of Se, Sp, Acc and Auc. As for the LBF and LGDF models, the latter is more accurate than the former, with greater by 0.023, 0.016 and 0.010 for Se, Acc and Auc, respectively.

**Table 1:** Segmentation performance of different models in terms of Se, Sp, Acc and Auc.

Method	Se	Sp	Acc	Auc
CV	0.617	0.652	0.589	0.635
LBF	0.704	0.958	0.921	0.831
LGDF	0.727	0.952	0.937	0.840
MGDF	0.758	0.965	0.952	0.862

To evaluate their statistical differences, paired t-test on the final performance measures (*i*.*e*., Acc and Auc) is implemented based on all of segmentation results. The mean and standard deviation of Acc is 0.589±0.087, 0.917±0.012, 0.933±0.010 and 0.954±0.009 for the CV, LBF, LGDF and MGDF models, respectively. The difference between them is statistically significant with p < 0.002. There are also statistically significant differences in terms of Auc according to the mean and standard deviation of these models for the same p value. This means that phase-based vessel enhancement contributes significantly to the final performance results. This further confirms that in image segmentation, other texture information besides image intensities need also be considered in both global and local image regions, because they in general play an important role in identifying small vessels from image regions where intensity inhomogeneity is severe.

Although our proposed model outperforms these typical active contour models in performance, it is necessary to compare with other state-of-the art vessel segmentation methods. For this propose, several widely used vessel segmentation methods [37–41] are chosen, together with their publicly available results based on STARE database. The segmentation results are seen in Table 2, which clearly shows that our proposed method is has the first highest values for Se and Auc, and third for Acc. This demonstrates that our method can compete with these methods according to the final measures (*i*.*e*., Acc and Auc).

**Table 2:** The performance of different methods in terms of in terms of Se, Sp, Acc and Auc.

Model	Se	Sp	Acc	Auc
You et al [37]	0.726	0.975	0.949	0.851
Marin et al [38]	0.694	0.981	0.952	0.838
Mendonca et al [39]	0.699	0.973	0.944	0.836
Matinez et al [40]	0.750	0.956	0.941	0.853
Bankhead et al [41]	0.758	0.950	0.932	0.854
MGDF	0.758	0.965	0.944	0.862

## Discussion

Experimental results demonstrate that our model is competent for the task of vessel segmentation, and outperforms some existing typical vessel segmentation methods, but it is necessary to discuss the influences of the whole weighting parameters in our model, most of which have been deeply analyzed in previous studies [26, 42]. Hence, our focus is placed on the analyses of the robustness of our model for initial placement and image noises, the parameters $\lambda_{i}^{I},\lambda_{i}^{V},i=1,2$ for intensity- and vesselness-based terms, and *η* for the ϒ-neighborhood regularization term respectively.

### Robustness to Initialization and Image noises

**Fig 5:**
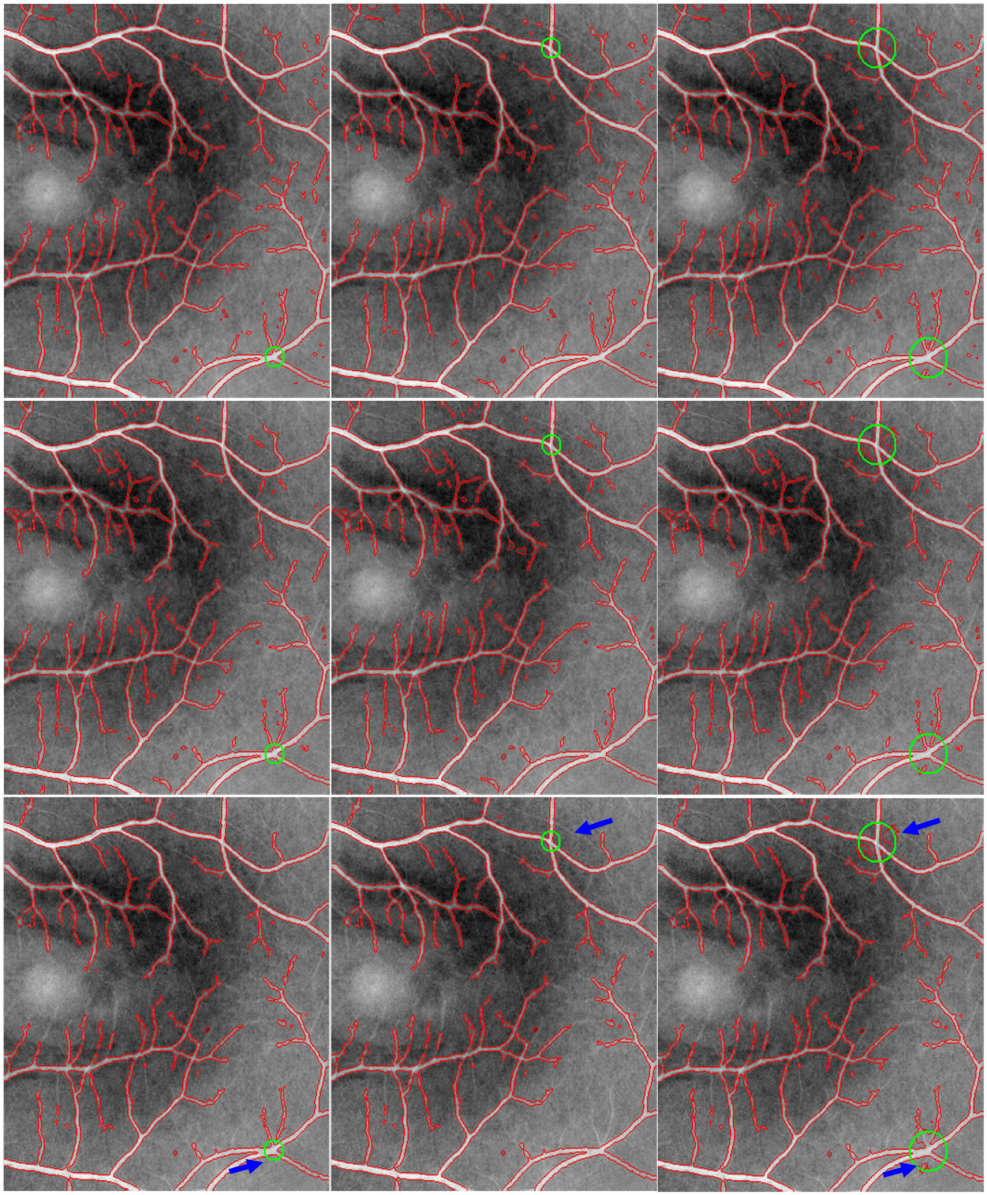
The segmentation results of our model on a randomly chosen image with different initializations highlighted by green circles. The image is corrupted by additive Gaussian noise with standard deviations 1, 3 and 5, showed in different rows.

In order to further analyze the robustness of our model to different initial placements and image noises, our model is evaluated based on one randomly chosen image. This image is corrupted by commonly used additive Gaussian noise with different standard deviations presented in different columns. In this section, these deviations are set to 1, 3 and 5, respectively, which are large enough relative to the width of blood vessels. Based on these noise-corrupted images, the segmentation results of our model are obtained using different initial curve placements underlined by green circles, as shown in Fig 5. According to these segmentation results, we can find that the segmentation performances of our model reduces as standard deviations increase, but our model can still extract most of vessels regardless of different initializations, as in the first two rows in Fig 5. Besides, the two rows also show that there exist segmentation differences in the neighborhood of small vessels which are severely corrupted by image noises. However, these differences are small because these positions play a relatively small role in clinical applications. This means that our model is competent for clinical applications in presence of intensity inhomogeneity, image noises and the initial curve placements. Hence, our model is to some extent insensitive to image noises and the initial curve placements.

Although the influences of the initial curve placements on our model is small, the size of initial curves can influence the segmentation performances of our model in the neighborhood of initial curves, as shown in the last row in Fig 5, where the bigger initial green circlers are, the more the isolated connected components are, ignoring the impact of image noises. This may be caused partly by the size of the desired objects. Therefore, it is desirable to initialize curves in accordance with the size of desired objects.

### The parameters $\lambda_{i}^{I}$ and $\lambda_{i}^{V}$

**Fig 6:**
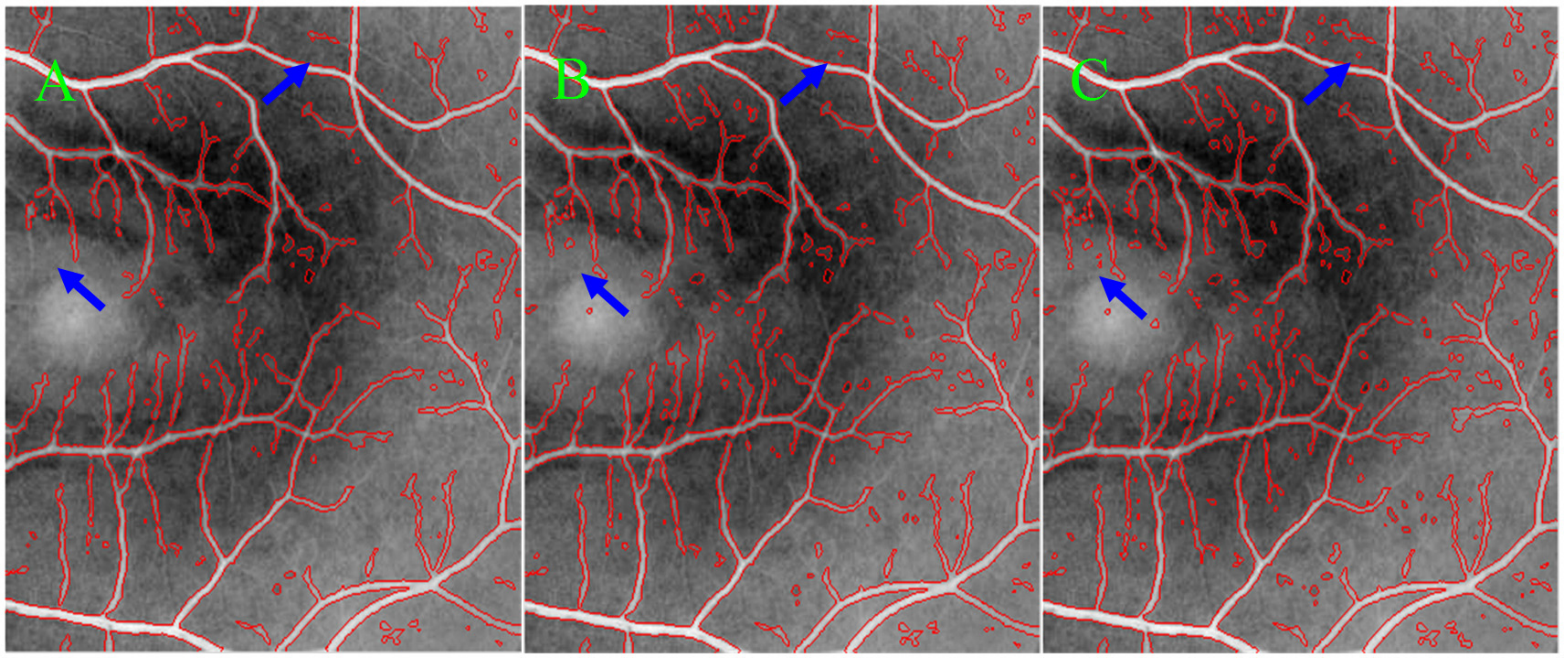
The Comparison of the segmentation results for different values $\lambda_{1}^{V}$ in the MGDF model. This value is set to 1.0, 1.05 and 1.1 for (A), (B) and (C), respectively.

The parameters $\lambda_{1}^{I}$ and $\lambda_{1}^{V}$ are the weights of image regions inside *C*; while $\lambda_{2}^{\text{I}}$ and $\lambda_{2}^{V}$ for image regions outside. They are in general recommended about 1.0 according to the previous studies [42], but adjusted to yield better results according to the natures of intensity images and vesselness maps. In segmentation experiments, these four parameters are set $\lambda_{1}^{I}=\lambda_{1}^{V}=1.05$, $\lambda_{2}^{I}=\lambda_{2}^{V}=1.0$, which means that image intensities and the vesselness values of vesselness map have the same effects on the motions of curve contours. When the intensity inhomogeneity is severe, the intensity-based terms play a weak role in attracting the contour toward object boundaries. In such case, we should choose relatively large $\lambda_{i}^{V}$ to stress the roles of vesselness maps for the extraction of small vessels in the regions with severe intensity inhomogeneity. However, too large $\lambda_{i}^{V}$ may lead to the extraction of isolated connected components due to the natures of retinal images and vesselness maps, as shown in Fig 6, where blue arrows specified the differences between segmentation results with different values of $\lambda_{1}^{V}$, *i*.*e*., 1.0, 1.05 and 1.1 for Fig 6(A) and 6(B), respectively. Hence, it is a good tradeoff between small vessels and isolated components by setting $\lambda_{1}^{I}=\lambda_{1}^{V}=1.05$ and $\lambda_{2}^{I}=\lambda_{2}^{V}=1.0$.

### The parameter η

The parameter *η* is used to control the influences of isolated connected components in segmented images so as to keep fine details of the desired boundaries. When setting a large value to *η*, it, to some degree, can prevent the isolated components, but also reduce the ability of identifying small vessels due to the presence of intensity inhomogeneity and image noises. In addition, this ability is also influenced greatly by the vesselness-based weighting parameters according to the analysis above, which suggests that it is complex to work out the optimal value for *η* for the correct extraction of desired objects, along with minimal amount of the undesired isolated components. In this paper, the parameter *η* is chosen to 1.0 as a tradeoff between them.

## Conclusions

In this paper, a novel region-based active contour model is proposed and employed to segment vessels in retinal images, which takes image intensities and ‘vesselness values’ as two independent random variables with different means and variances, and then uses the two variables to construct a multi-feature Gaussian distribution fitting energy so as to improve the segmentation performances of the LGDF model. The novel model is evaluated and compared with the existing typical active contours (*i*.*e*., the CV, LBF and LGDF models) based on publicly available retinal datasets, the experimental results demonstrate that our model outperforms these typical region-based models in terms of sensitivity, specificity, accuracy and the area under a receiver operating characteristic curve.

## Supporting Information

S1 File Permission from the original copyright holder for STARE database. It permits us to use any materials posted at the cited web site http://www.ces.clemson.edu/~ahoover/stare/ for this paper.
(DOC)
