# Supplementary material for: Active Contours Driven by Multi-Feature Gaussian Distribution Fitting Energy with Application to Vessel Segmentation
Source: PLoS One. 2015 Nov 16;10(11):e0143105. doi: 10.1371/journal.pone.0143105 (PMC4646657; doi:10.1371/journal.pone.0143105)
Supplement: S1 File — It permits us to use any materials posted at the cited web site http://www.ces.clemson.edu/~ahoover/stare/ for this paper. (DOC) [file pone.0143105.s001.doc]

**Written Permission**

Dear article authors,

I provide permission to use any materials posted at the cited web site http://www.ces.clemson.edu/~ahoover/stare/, to publish your research paper (*i.e.,* Active contours driven by multi-feature Gaussian distribution fitting energy with application to vessel segmentation) in "PLOS ONE" under the specific Creative Commons Attribution License (CCAL), CC BY 4.0.

Adam Hoover, PhD

Professor

Electrical & Computer Engineering Department

Clemson University

Clemson, SC 29634-0915

ahoover@clemson.edu

http://www.ces.clemson.edu/~ahoover
